# Age-disparate sex and HIV risk for young women from 2002 to 2012 in South Africa

**DOI:** 10.7448/IAS.19.1.21310

**Published:** 2016-12-26

**Authors:** Meredith Evans, Kathryn Risher, Nompumelelo Zungu, Olive Shisana, Sizulu Moyo, David D Celentano, Brendan Maughan-Brown, Thomas M Rehle

**Affiliations:** ^a^ Department of Anthropology, York University, Toronto, Canada; ^b^ Department of Epidemiology, Johns Hopkins Bloomberg School of Public Health, Baltimore, MD, USA; ^c^ HIV/AIDS, STIs and TB (HAST) Programme, Human Sciences Research Council, Cape Town, South Africa; ^d^ Evidence Based Solutions, Cape Town, South Africa; ^e^ Department of Psychiatry and Mental Health, University of Cape Town, Cape Town, South Africa; ^f^ Southern Africa Labour and Development Research Unit (SALDRU), University of Cape Town, Cape Town, South Africa; ^g^ School of Public Health and Family Medicine, University of Cape Town, Cape Town, South Africa

**Keywords:** Age-disparate sex, HIV, intergenerational sex, South Africa, women, sexual behaviour

## Abstract

**Introduction**: Age-disparate sex has long been considered a factor that increases HIV risk for young women in South Africa. However, recent studies from specific regions in South Africa have found conflicting evidence. Few studies have assessed the association between age-disparate partnerships (those involving an age gap of 5 years or more) and HIV risk at the national level. This study investigates the relationship between age-disparate sex and HIV status among young women aged 15–24 in South Africa.

**Methods**: Nationally representative weighted data from the 2002, 2005, 2008, and 2012 South African National HIV Surveys were analysed for young women aged 15–24 years using bivariate analyses and multiple logistic regressions.

**Results**: After conducting multiple logistic regression analyses and controlling for confounders, young women with age-disparate partners had greater odds of being HIV positive in every survey year: 2002 (aOR = 1.74, 95%CI: 0.81–3.76, p = 0.16); 2005 (aOR = 2.11, 95%CI: 1.22–3.66, p < 0.01); 2008 (aOR = 2.02, 95%CI: 1.24–3.29, p < 0.01); 2012 (aOR = 1.53, 95%CI: 0.92–2.54, p < 0.1). The odds of being HIV positive increased for each year increase in their male partner’s age in 2002 (aOR = 1.10, 95%CI: 0.98–1.22, p = 0.11), 2005 (aOR = 1.10, 95%CI: 1.03–1.17, p < 0.01), 2008 (aOR = 1.08, 95%CI: 1.01–1.15, p < 0.05), and 2012 (aOR = 1.08, 95%CI: 1.01–1.16, p < 0.05). Findings were statistically significant (p < 0.1) for the years 2005, 2008, and 2012.

**Conclusions**: Our findings suggest that age-disparate sex continues to be a risk factor for young women aged 15–24 in South Africa at a national level. These results may reflect variation in HIV risk at the national level compared to the differing results from recent studies in a demographic surveillance system and trial contexts. In light of recent contradictory study results, further research is required on the relationship between age-disparate sex and HIV for a more nuanced understanding of young women’s HIV risk.

## Introduction

Young women carry a disproportionate burden of the HIV epidemic, accounting for one in four new HIV infections in sub-Saharan Africa [1]. In South Africa, national household HIV prevalence and behavioural surveys conducted triennially since 2002 have consistently demonstrated that HIV prevalence and new HIV infections remain elevated among young women [2–5].

High rates of HIV infection in young women have been attributed to the interplay between biological, socio-behavioural, and epidemiological factors. Having sexual relationships with men five years and older, referred to as age-disparate partnerships, has been identified as a contributing factor to the higher prevalence of HIV in young women in southern Africa [6–10]. The South African national household HIV surveys consistently find large proportions (around one third) of women in age-disparate partnerships, which highlights the potential importance of these partnerships for HIV risk.

Young women are thought to have greater likelihood of exposure to HIV when they have sex with older men compared to their male peers since older men have a greater burden of sexually transmitted infections including HIV [9–11]. In South Africa, the prevalence of HIV among men 25–54 years old was significantly higher than men 15–24 years old (20.9% vs 2.9%) in 2012 [5].

Furthermore, unequal gender, race, and economic power dynamics amplified by age differences can lead to greater risk-taking behaviour among young women with age-disparate male partners, such as condomless sex [9,12–14], earlier age of first sex [9,15,16], increased number of lifetime partners [9], and multiple sexual partnerships [15,17]. In addition, unequal power dynamics in age-disparate relationships make it difficult for women to negotiate safer sex and condom use with older partners [7,12], and young women’s assertive behaviour in sexual relationships may result in physical or sexual violence from male partners [18–20].

Despite the existing evidence that suggests age-disparate sex is an HIV risk factor for young women, two recent longitudinal studies did not find a relationship between age-disparate sex and HIV incidence for young women in two South African contexts: a study conducted from 2003 to 2012 in a demographic surveillance system site within the district uMkhanyakude, Kwa-Zulu-Natal, a small rural community [21] and a clinical trial study (VOICE) conducted with participants in three urban areas of Durban, KwaZulu-Natal; Johannesburg, Gauteng; and Klerksdorp, North West province from 2009 to 2012 [22]. One explanation for these contradictory findings is that the specific contexts investigated differ from other settings, including the general national context. The VOICE trial was restricted to women willing to use highly effective forms of contraception, and the data analysis was limited further to women who reported a primary partner at enrolment [22]. The surveillance study setting in the district of uMkhanyakude is marked by extreme deprivation, high levels of migration, and extremely high levels of HIV prevalence and infection rates [21]. As a small, cohesive rural community in a long-term surveillance study, it is possible that young women may be more accurately informed of the risks of older male partners [21]; this is supported by recent evidence that suggests risk of age-disparate partnerships is amplified for young women in urban compared to rural areas [17]. Finally, both the trial and surveillance contexts could be influenced by the frequency of HIV testing and other HIV services, and the increased awareness of risk through long-term interaction with researchers [23,24]. Since the participants in both the surveillance and trial context may not be representative of the average population, it is unclear whether results are generalizable to other settings, including the national context.

Since the most recent national-level study investigating the relationship between age-disparate sex and HIV in South Africa is from 2003 [8], another explanation for the contradictory findings could be that the relationship between age-disparate sex and HIV has changed over time. The risk of age-disparate male partners may have decreased since 2003 following the scaling up of antiretroviral therapy (ART). Older HIV-positive men aged 25–49 are more likely to be on ART than younger men, and therefore may be less likely to transmit HIV [5,25]. Therefore, an updated analysis of age-disparate sex that is representative of the national context is necessary. In this study, we investigate the associations between age-disparate partnerships and HIV status among young women aged 15–24 years in South Africa from 2002 to 2012 using four nationally representative cross-sectional household HIV prevalence and behavioural surveys conducted in 2002, 2005, 2008, and 2012.

## Methods

### Data

The detailed methodology of the South African national HIV prevalence and behavioural household surveys has been described elsewhere [2–5]. In sum, national population-based household surveys were conducted using multistage stratified cluster sampling. A systematic probability sample of 15 households was drawn from each of 1000 enumeration areas (EAs) selected randomly from strata defined by locality type and province from the 2001 census EAs for the 2002 and 2005 surveys and with the updated 2007 master sample used for the 2008 and 2012 surveys. A detailed questionnaire soliciting information related to knowledge, attitudes, behaviours, and demographics was administered to participants. In 2005, 2008, and 2012, dried blood spot specimens collected by nurses through the finger-prick were tested anonymously for HIV antibodies using a testing algorithm with three different immunoassays (First EIA: Vironostika HIV uni-form II plus O, biomeriux, boxtel, the Netherlands; second EIA: Advia centaur XP, siemens medical solutions diagnostics, Tarrytown, NJ, USA; third EIA: Roche elecys 2010 HIV combi, roche diagnostics, Mannheim, Germany). The 2002 survey used oral transudate specimens for HIV antibody testing. Participants were asked to give informed consent (written or verbal in cases of illiteracy) before the interview and blood specimen collection. Participants under 18 years of age were required to provide written assent by means of a signature, with informed consent required of their parents or guardians in order for them to participate. The individual response rates for the questionnaire were 93.6% in 2002, 96.0% in 2005, 89.1% in 2008, and 89.5% in 2012 (with questionnaire non-response rates ranging from 4.0–10.9%). The individual response rates for HIV testing were 62.3% in 2002, 65.4% in 2005, 64.3% in 2008, and 67.5% in 2012 (with HIV testing non-response rates ranging from 32.5–37.7%). Data were collected, validated, adjusted for non-response and weighted for all survey years, and in 2008 and 2012 data were double captured and verified using CSPro [26]. Ethical approval for each survey was obtained from the Ethics Committee of the Human Sciences Research Council, South Africa, and Centres for Disease Control, USA.

Responses from female participants aged 15 to 24 who reported being sexually active in the past 12 months were included in the main analysis. Responses from male participants aged 15 to 34 who reported being sexually active in the past 12 months were included for HIV prevalence estimates only. The 2005, 2008, and 2012 surveys collected data on the age of women’s most recent sexual partners (up to three) in the past 12 months, whereas the 2002 survey contained only the details of current partners at the time of the survey. The 2012 survey explicitly identified partners’ sex thus allowing selection of male partners only for this analysis. As very few women reported female partners in the 2012 sample, the data for the 2002, 2005, and 2008 survey years are assumed to reflect young women in partnerships with men.

### Measures

Partner ages were assessed by asking participants the ages of their recent sexual partners. Based on the UNAIDS definition [27], the binary variable age-disparate sex identifies young women who reported at least one of their recent sexual partners as older by 5 years or more (=1), with the comparator group identifying young women who reported no partner 5 years or more older (=0). Young women with partners more than 5 years younger were rare (under 0.5% reported partners 5 or more years younger across all four surveys) and were coded as age-similar since these partnerships are not assumed to carry additional risk for young women. Because of low numbers of young women who reported partners more than 10 years older (under 10.0% reported partners 10 or more years older across all four surveys), the analysis for age-disparate sex could not consider differences between partnerships with an age gap of 5–9 years to those with a 10 year or more age gap. Additionally, a continuous measure of aged-disparate partnerships was created as the difference in years between the respondent and her partner. To minimize the influence of outliers in regression analyses using this continuous measure, respondents with a partner 2 or more years younger were assigned the value −2 and respondents with a partner 16 or more years older were assigned the value +16 (under 3.5% of partners were younger by 2 years or more and 2.5% of partners were older by 16 years or more across all four survey years). Notably, very few women reported having multiple sexual partners (less than 6% of women reported more than one sexual partner in any survey year); therefore, the analysis is largely based on the respondent’s most recent partner.

For our regression analyses we create several variables to control for potential confounding factors. In addition to basic demographic characteristics (age, race, marital status, and locality type) we included a measure of employment status given the association between wealth and both HIV status [28–31] and the formation of age-disparate partnerships [6,7,32–35]. We also created a set of sexual behaviour measures for each survey year. First, multiple sexual partners (two or more sexual partners in the past 12 months) was included as it is associated with HIV risk [4,5]) and the more partners one has the greater the likelihood of having any age-disparate partnerships. Second, condom use at last sexual intercourse was considered because of the association between condomless sex and HIV risk [4,5] as well as age-disparate partnerships [9,12–14] which could in part be explained by evidence that suggests older men seek younger women partners since they believe them to be HIV free [9,35,36] and younger women seek older men as they also believe them to be safer [37]. Third, age of first sex was included as it is associated with increased risk of HIV for young people [38,39] and the formation of age-disparate partnerships [9,11]. The marital status variable is measured in two different ways: firstly, married compared to unmarried for comparison across all survey years, and, secondly, married compared to cohabitating and single for further analysis in the 2012 survey year. In 2012 three additional variables were available and included for further analysis: number of lifetime sexual partners; perceived partner concurrency (obtained from the question: Do you think your most recent sexual partner had other sexual partners in the past 12 months?) as concurrency may be associated with age-disparate partnerships [40]; and individual income in the past month.

### Analysis

First, HIV prevalence rates are presented for all survey years for sexually active young women aged 15–24, young women who reported age-similar partners and those that reported age-disparate partners. HIV prevalence data is also presented for sexually active men aged 15–24 and men aged 25–34. We selected these age ranges for men as the majority of young women’s age-similar partners will be between 15–24 years old and the majority of young women’s age-disparate partners will be between 25–34 years old. The upper bound was 34 years old as the majority (95%+) of all young women’s partners in our data were younger than 35 years old. Sociodemographic characteristics of young women in each survey are then presented. Simple logistic regressions were conducted to assess bivariate relationships between age-disparate sex, sociodemographic characteristics, sexual behaviour and HIV status. Multiple logistic regressions (with 95% confidence intervals and adjusted odds ratios displayed) were conducted using 2002, 2005, 2008 and 2012 survey data to investigate the relationship between age-disparate sex and HIV status for young women aged 15–24, controlling for factors that could potentially influence both HIV status and age-disparate partnerships (model A). The 2012 models were repeated to include additional potential confounders available in that survey year (model B). All analyses were conducted twice, firstly with age-disparate sex as a categorical variable and secondly with a continuous measure of age difference in years. STATA version 12 was used for analysis. Complete case analyses were conducted for all models, restricted to young women aged 15-24 who reported being sexually active in the past 12 months and who provided blood specimens for HIV testing. We excluded participants with missing data on any variable used in our analyses (selective refusal), resulting in the following missing data: 6.8% in 2012, 5.4% in 2008, 8.2% in 2005 and 20.4% in 2002. All analyses were weighted to account for the multi-stage cluster sampling design and for non-response to HIV testing.

## Results

**Figure 1.**
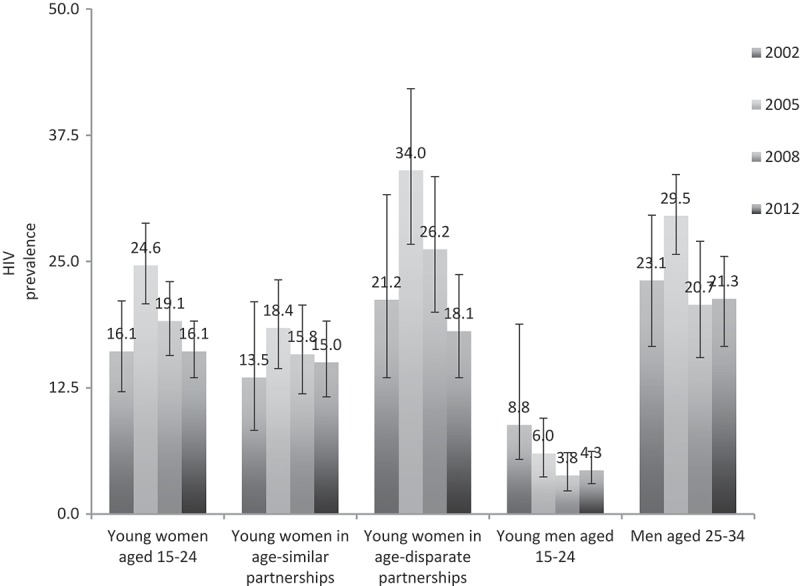
HIV prevalence for young women aged 15–24 years and men aged 15–34, South Africa 2002, 2005, 2008, and 2012.

HIV prevalence for sexually active young women aged 15-24 years was 16.1% (N = 563) in 2002, 24.6% (N = 1089) in 2005, 19.1% (N = 877) in 2008, and 16.1% (N = 1490) in 2012 (Figure 1). HIV prevalence was consistently greater among young women with age-disparate partners (5 years or older) compared to young women with age-similar partnerships; however, differences were only statistically significant below the 5% level for the 2005 (34.0% vs 18.4%, p < 0.01) and 2008 (26.2% v 15.8%, p < 0.01) survey years. HIV prevalence for sexually active young men aged 15-24 (i.e. the men more likely to be in the age-similar partnerships) was 8.8% (N = 446) in 2002, 6.0% (N = 714) in 2005, 3.8% (N = 801) in 2008, and 4.3% (N = 1259) in 2012. HIV prevalence for men aged 25-34 was much higher, 23.1% (N = 472) in 2002, 16.9% (N = 724) in 2005, 20.7% (N = 531) in 2008, and 21.2% (N = 1746) in 2012. For additional detail on HIV prevalence stratified by sex and age, see the national HIV study reports [2–5].

Sociodemographic characteristics of sexually active young women aged 15–24 years in the 2002, 2005, 2008, and 2012 survey years are presented in Table 1. Most women were aged 20–24 years old, black African, single or unmarried, living in urban formal or rural informal areas, unemployed, and had secondary level education, whereas just over one quarter were students. Close to one-third of participants reported at least one age-disparate partner in all survey years: 39.9% (N = 495) in 2002; 39.3% (N = 1063) in 2005; 31.3% (N = 877) in 2008; and 36.2% (N = 1490) in 2012. Sexual behaviours reported by sexually active young women aged 15-24 years in the 2002, 2005, 2008, and 2012 survey years are presented in Supplementary digital content Table S1.

**Table 1.** **Sociodemographics of young women aged 15** **–** **24 years, South Africa 2002, 2005, 2008, and 2012.**

	2002	2005	2008	2012
	N = 560	N = 1083	N = 903	N = 1496
	n (%)	n (%)	n (%)	n (%)
**Age (years)**	N = 495	N = 1063	N = 877	N = 1490
15–19	152 (29.6)	276 (24.6)	271 (34.3)	453 (32.0)
20–24	343 (70.4)	787 (75.4)	606 (65.7)	1037 (68.0)
**Race**	N = 495	N = 1061	N = 877	N = 1489
Black African	350 (85.5)	803 (88.5)	661 (88.9)	1116 (86.6)
Non-Black African	145 (14.5)	258 (11.5)	216 (11.1)	373 (13.4)
**Marital Status**	N = 488	N = 1062	N = 873	N = 1480
Single	–	844 (81.9)	713 (83.6)	1142 (78.2)
Married	68 (13.9)	144 (11.1)	95 (9.0)	128 (7.4)
Cohabitating	–	74 (7.0)	65 (7.3)	210 (14.4)
Unmarried	420 (86.1)	–	–	–
**Locality Type**	N = 495	N = 1063	N = 877	N = 1490
Urban Formal	254 (43.0)	461 (37.4)	400 (43.2)	717 (51.2)
Urban Informal	60 (8.9)	171 (10.1)	184 (12.7)	213 (8.2)
Rural Informal	131 (41.8)	310 (43.9)	228 (38.0)	423 (37.0)
Rural Formal	50 (6.4)	121 (8.5)	65 (6.0)	137 (3.7)
**Employment**	N = 472	N = 1056	N = 875	N = 1457
Unemployed	278 (61.8)	617 (60.6)	482 (55.8)	810 (53.1)
Student	111 (26.1)	264 (26.7)	247 (32.4)	412 (31.8)
Employed	83 (12.1)	175 (12.6)	146 (11.8)	235 (15.1)
**Education**	N = 491	N = 1058	N = 873	N = 1376
Primary or less	80 (15.7)	164 (14.7)	90 (7.6)	149 (8.4)
Secondary	378 (76.3)	826 (79.8)	724 (85.0)	1159 (85.7)
Tertiary	33 (8.0)	68 (5.4)	59 (7.4)	68 (5.9)

Bi-variate associations between HIV status and control variables are displayed in Supplementary digital content Table S2. Women who reported any age-disparate partner had significantly greater odds of prevalent HIV infection compared to those with only age-similar partner(s) in 2005 (OR = 2.28, p < 0.01) and 2008 (OR = 1.88, p < 0.01). Analyses using the continuous measure of age difference in years between partners found a significant and positive association in 2005 (OR: 1.10, p < 0.01), 2008 (OR: 1.07, p < 0.05), and 2012 (OR: 1.08, p < 0.05).

The findings from the multiple logistic regression analyses of the association between age-disparate sex and HIV status for young women aged 15–24 in 2002, 2005, 2008, and 2012 are presented in Table 2. Having had age-disparate partnerships significantly increased the odds of being HIV positive for young women aged 15–24 in 2005 (aOR = 2.11, p < 0.01) and 2008 (aOR = 2.02, p < 0.01) compared to having had age-similar partnerships. After repeating the model for 2012 including three additional control variables, the relationship between our binary measure of age-disparate sex and HIV status was found to be statistically significant at the 10% level (Model 2012 (B): aOR = 1.53, p < 0.1).

**Table 2.** **Multiple regression analyses showing associations between age-disparate sex (5+ years older) and HIV status for young women aged 15** **–** **24 years, South Africa 2002, 2005, 2008, and 2012.**

	2002 (A) N = 446	2005 (A) N = 994	2008 (A) N = 854	2012 (A) N = 1394	2012 (B) N = 1257
	aOR (95%CI)	aOR (95%CI)	aOR (95%CI)	aOR (95%CI)	aOR (95%CI)
**Age-disparate 5+ yrs (vs age-similar ≤4 yrs)**	1.74 (0.81–3.76)	2.11*** (1.22–3.66)	2.02*** (1.24–3.29)	1.28 (0.78–2.10)	1.53* (0.92–2.54)
**Control variables**	YES	YES	YES	YES	YES

Note: * = p < 0.10; ** = p < 0.05; and *** = p < 0.01

Control variables included in models (A): age, race, marital status, locality, employment status, condom use at last sex, age of first sex, and multiple sexual partnerships.

Control variables included in models (B): age, race, marital status, locality, employment status, condom use at last sex, age of first sex, lifetime partners, think partner had multiple sexual partners, and personal income last month.

See Supplementary Digital Content for the full models, showing the coefficients for all variables.

All analyses were adjusted to account for the complex study design (i.e. stratification and clustering) and for non-response (by using weighted data).

Table 3 presents the findings from the multiple logistic regression analyses of the association between the continuous variable of partner age difference in years and HIV status for young women aged 15–24 in each survey year. Results indicate that young women had significantly increased odds of testing HIV-positive for each additional year that their male partners were older than them in 2005 (aOR = 1.10, p < 0.01), 2008 (aOR = 1.08, p < 0.05), and 2012 aOR = 1.08, p < 0.05). The model for 2012 was repeated controlling for the three additional control variables available in the 2012 survey year, with similar results (Model 2012 (B): aOR = 1.08, p < 0.05).

**Table 3.** **Multiple regression analyses showing associations between age difference in years between partners (continuous) and HIV status for young women aged 15** **–** **24 years, South Africa 2002, 2005, 2008, and 2012.**

	2002 (A) N = 446	2005 (A) N = 994	2008 (A) N = 854	2012 (A) N = 1394	2012 (B) N = 1257
	aOR (95%CI)	aOR (95%CI)	aOR (95%CI)	aOR (95%CI)	aOR (95%CI)
**Age difference in years (continuous)**	1.10 (0.98–1.22)	1.10*** (1.03–1.17)	1.08** (1.01–1.15)	1.08** (1.01–1.16)	1.08** (1.01–1.15)
**Control variables**	YES	YES	YES	YES	YES

Note: * = p < 0.10; ** = p < 0.05; and *** = p < 0.01

Control variables included in models (A): age, race, marital status, locality, employment status, condom use at last sex, age of first sex, and multiple sexual partnerships.

Control variables included in models (B): age, race, marital status, locality, employment status, condom use at last sex, age of first sex, lifetime partners, think partner had multiple sexual partners, and personal income last month.

See Supplementary Digital Content for the full models, showing the coefficients for all variables.

All analyses were adjusted to account for the complex study design (i.e. stratification and clustering) and for non-response (by using weighted data).

The full models of the multiple regression analyses are presented in the Supplementary digital content (Tables S3 and S4 in Additional file 1). Sensitivity analyses showed that the results remained consistent when the analysis was restricted to most recent partner only (Supplementary digital content, Tables S5, S6 and S7 in Additional file 1). When restricted to black African women only (Supplementary digital content, Tables S8, S9, and S10 in Additional file 1), the relationship between age-disparate sex and HIV status increased substantially in 2002 and was statistically significant (aOR = 2.39, p < 0.05) whereas in 2012 the relationship decreased and was not statistically significant (aOR = 1.24, p = 0.39). The relationship between partner age difference in years and HIV status also increased in 2002 when restricted to black African women only and was statistically significant (aOR = 1.21, p < 0.01), whereas the results from other survey years remained similar to the original findings.

## Discussion

In South Africa, HIV prevention efforts are particularly important among young women as HIV prevalence and incidence is much higher among young women than their male counterparts [4,5]. Consequently, improving our understanding of risk factors among young women is crucial. This study provides nationally representative evidence that in 2005, 2008, and 2012, age-disparate sex was significantly associated with elevated HIV infection risk among young women aged 15-24 years old in South Africa. In 2005 and 2008 having had an age-disparate partner 5+ years older more than doubled young women’s odds of being HIV positive, and in 2005, 2008, and 2012 young women’s odds of being HIV positive increased for each year increase in their partner’s age. While positive, the lack of statistical significance between partner age and HIV-status in the 2002 data is likely, in part, due to the small sample size in that particular survey. Although the relationship between age-disparate sex (using the binary measure) and HIV prevalence remained relatively consistent across the four survey years, the relationship was weaker in 2012. This could potentially be linked to the scaling up of ART in recent years which is better accessed by older HIV-positive men aged 25-49 than younger HIV-positive men [5,25], who are more likely to be recently infected, not on ART, and highly infectious.

Overall, this study supports the hypothesis that, at a national level, age-disparate sex has been and may continue to be a risk factor for HIV infection among young women 15-24 years old in South Africa. These findings are in line with previous research [8], and are supported by the recent data based on molecular epidemiological evidence suggesting that age-disparate sex increases young women’s risk of HIV [41]. Based on HIV phylogenetic analyses, this study showed that the high HIV prevalence in young women is driven by sex with older men (on average 8.7 years older) who themselves had partners who had HIV prevalence rates greater than 60% [41]. However, this recent phylogenetic study and our results differ from those in a recent surveillance study [21] and a clinical trial [22] which found no association between age-disparate partnerships and HIV incidence among young women. Differences between study results may reflect variation in HIV risk in community contexts compared to the national context, and differences in study sample and design. It may also reflect differences in the outcomes used: HIV incidence compared to HIV prevalence. The temporal sequence of causation is more difficult to interpret in prevalence than in incidence studies.

Several potential limitations of this study should be considered. Social desirability and recall bias might have influenced self-reported measures of sexual behaviour, and there is the potential for error in reporting of partnerships [42] and partner’s age [43]. Although we found positive associations between HIV status and age-disparate sex for young women, causality cannot be inferred due to the cross-sectional nature of the study. Furthermore, transactional sex is a potential confounding factor in the relationship between age-disparate sex and HIV; however, since transactional sex was not adequately measured in any survey year we were unable to include it in our analysis. Additional potential confounding factors may exist that were not measured or included in the models. The generalizability of the 2002 results may have been affected by excluding participants with missing data from the analyses. While our results suggest that age-disparate partnerships increase risk for young women aged 15–24, this association may not hold for older women, especially since the proportion of HIV positive men increases with age only until age 40 after which it subsequently declines [5]. Further research is needed to explore whether age-disparate sex is a risk factor among older women.

As our results suggest that age-disparate sex is a risk factor for HIV infection among young women aged 15-24 years, interventions to reduce the risk that age-disparate partnerships pose for young women may be warranted. However, caution in the interpretation of our findings and how they are used to inform policy and programmes is recommended. In recent years, it has been questioned whether prevention messages discouraging age-disparate relationships should continue [44–48] and whether such messages risk stigmatizing young women who engage in these relationships [49]. However, the media continues to sensationalize “sugar daddies” and “blessers” with individual narratives about vulnerable young women [50–52] and the current intervention strategy appears to continue the same approach of simply discouraging young women from engaging in such relationships [53,54]. Instead, we need research and intervention strategies that seek to understand and address the underlying conditions that make young women especially at risk in sexual relationships with older men, for instance financial, educational, and social vulnerability. Transactional relationships have been long understood as a primary motivating factor for young women to engage in sexual partnerships with older men [7,24,32], since economic asymmetry gives older men even more power in negotiating sex with younger women who are financially vulnerable [15,55,56]. A recent intervention providing cash transfers to increase young women’s economic independence had success in reducing the risk of HIV infection [57]. Expanding such interventions to address young women’s financial, educational, and social vulnerability on a broader scale could have the potential not only to reduce the HIV risk of age-disparate partnerships but also to increase young women’s general wellbeing in South Africa. Biomedical research and interventions such as access to pre-exposure prophylaxis (PrEP) should also be prioritized for young women and girls.

Finally, the majority of research studies, prevention strategies, and media attention on the topic of age-disparate sex have thus far focused attention on young women in these relationships, instead of their older male partners. This approach risks not only further stigmatizing young women but also overlooking the role of men. Further investigation is necessary to understand the profiles of older men in sexual relationships with young women, who the “sugar daddies” (and “blessers”) are and whether their power in relationships with younger women differs depending on their relative age, education, or socioeconomic status. The motivations that drive older men to seek out young women in sexual relationships with especially skewed power relations need to be better understood. Moreover, efforts must be scaled-up to screen men for HIV and initiate HIV positive men on ART, as well as encourage men to undergo medical male circumcision and use condoms.

## Conclusion

In conclusion, our study provides evidence to support the continued identification of age-disparate sex as a risk factor at the national level for young women in South Africa. In light of recent contradicting study results, additional research is required to investigate the role that age-disparate partnerships play in young women’s HIV risk. Specifically, there is a need for further community level longitudinal and qualitative research in different areas across the country for a more nuanced understanding of age-disparate sex and young women’s HIV risk.

## Supplementary Material

Supplementary material

## Figures and Tables

**Table S1.** Sexual behaviour reported by young women aged 15–24 years, South Africa 2002, 2005, 2008, and 2012.

	2002 n (%)	2005 n (%)	2008 n (%)	2012 n (%)
**Condom use at last sex**	N = 550	N = 1072	N = 897	N = 1474
Yes	250 (45.4)	530 (53.0)	546 (62.7)	772 (50.6)
No or do not know	300 (54.6)	542 (47.0)	351 (37.3)	702 (49.4)
Consistent condom use with most recent partner*			N = 886	N = 1480
Every time	-	-	428 (50.7)	558 (36.9)
Almost every time	-	-	55 (5.0)	70 (6.9)
Sometimes	-	-	205 (24.1)	313 (22.0)
Never	-	-	198 (20.1)	539 (34.1)
**Age at first sex**	N = 544	N = 1044	N = 890	N = 1776
Aged ≤15	112 (20.7)	186 (15.5)	156 (18.5)	305 (15.5)
Aged 16+	432 (79.3)	858 (84.5)	734 (81.5)	1471 (84.5)
**Multiple sexual partnerships**	N = 557	N = 1063	N = 903	N = 1497
1 partner	507 (91.4)	1002 (94.1)	840 (93.5)	1376 (91.7)
2+ partners	50 (8.6)	61 (5.9)	63 (6.5)	121 (8.3)
**Number of lifetime partners**				N = 1446
1 lifetime partner	-	-	-	624 (43.5)
2 lifetime partners	-	-	-	369 (25.7)
3–4 lifetime partners	-	-	-	326 (23.2)
5+ lifetime partners	-	-	-	127 (7.5)
**Think partner had other sexual partners in past year**				N = 1454
Yes or do not know	-	-	-	738 (55.1)
No	-	-	-	716 (44.9)

*Consistent condom use was not measured in 2002 or 2005. Condom use at last sex was therefore included in the multiple regression analyses because it was measured in all survey years and because the measure of condom use at last sex is less subject to recall bias than consistent condom use.

**Table S2.** Bivariate regression analyses showing associations between select sociodemographic and sexual behaviours with HIV status for young women aged 15–24 years, South Africa 2002, 2005, 2008, and 2012.

	2002 OR (95% CI)	2005 OR (95% CI)	2008 OR (95% CI)	2012 OR (95% CI)
Age-disparate sex 5+ yrs older (vs ≤4yrs)	1.73 (0.86–3.45)	2.28*** (1.41–3.69)	1.88*** (1.17–3.03)	1.25 (0.77–2.02)
Age difference in years (continuous)	1.08 (0.98–1.19)	1.10*** (1.04–1.16)	1.07** (1.00–1.14)	1.08** (1.02–1.15)
*Sociodemographics*				
Age	1.09 (0.94–1.26)	1.19*** (1.07–1.32)	1.20*** (1.08–1.33)	1.15*** (1.05–1.25)
Non-black African (vs black African)	0.56 (0.19–1.65)	0.05*** (0.02–0.15)	0.12*** (0.05–0.29)	0.06*** (0.02–0.15)
Married (vs unmarried)	0.81 (0.28–2.34)			
Marital status (ref = single)				
Married		0.32*** (0.15–0.67)	0.47* (0.19–1.14)	0.43 (0.14–1.29)
Cohabitating		0.74 (0.33–1.65)	1.29 (0.59–2.80)	1.31 (0.70–2.46)
Locality (ref = urban formal)				
Urban informal	2.13* (0.87–5.18)	2.37*** (1.38–4.08)	1.93** (1.09–3.41)	1.81* (0.92–3.56)
Rural informal	0.52 (0.23–1.18)	1.10 (0.65–1.85)	1.02 (0.55–1.86)	1.56* (0.98–2.48)
Rural formal	1.50 (0.52–4.36)	1.07 (0.53–2.16)	1.90* (0.91–3.96)	0.79 (0.38–1.64)
Employment status (ref = unemployed)				
Student	0.45* (0.18–1.13)	0.88 (0.48–1.63)	0.47** (0.25–0.88)	0.46*** (0.26–0.81)
Employed	0.94 (0.36–2.45)	0.63 (0.33–1.21)	0.8 (0.40–1.60)	0.64 (0.34–1.20)
*Sexual Behaviour*				
Condom used at last sex (vs condom not used)	1.23 (0.62–2.45)	1.17 (0.76–1.82)	1.09 (0.66–1.79)	1.85*** (1.21–2.83)
Aged ≤15 at first sex (vs aged 16+)	0.87 (0.39–1.95)	0.98 (0.58–1.68)	1.13 (0.65–1.97)	1.18 (0.65–2.15)
Multiple sexual partners in past year (vs 1 partner)	1.97 (0.76–5.07)	2.74*** (1.36–5.54)	1.03 (0.43–2.44)	1.45 (0.78–2.72)
*Additional variables measured in 2012*				
Income past month >R4000 (vs ≤R4000)	–	–	–	0.13*** (0.04–0.44)
Number of lifetime partners (ref = 1 partner)				
2 lifetime partners	–	–	–	1.08 (0.61–1.91)
3–4 lifetime partners	–	–	–	2.15*** (1.22–3.78)
5+ lifetime partners	–	–	–	1.43 (0.62–3.26)
Think partner had other sexual partners (vs did not think)	–	–	–	2.48*** (1.54–3.99)

Note: *=p<0.10; **=p<0.05; and ***=p<0.01

All analyses were adjusted to account for the complex study design (i.e. stratification and clustering) and for survey non-response (by using weighted data).

**Table S3.** Multiple regression analyses showing associations between age-disparate sex (5+ years older) and covariates with HIV status for young women aged 15 to 24 years, South Africa 2002, 2005, 2008, and 2012.

	2002 (A) N=446	2005 (A) N=994	2008 (A) N=854	2012 (A) N=1394	2012 (B) N=1257
	aOR (95% CI)	aOR (95% CI)	aOR (95% CI)	aOR (95% CI)	aOR (95% CI)
Age-disparate 5+yrs older (vs ≤4yrs)	1.74 (0.81–3.76)	2.11*** (1.22–3.66)	2.02*** (1.24–3.29)	1.28 (0.78–2.10)	1.53* (0.92–2.54)
Age	1.04 (0.86–1.25)	1.23*** (1.09–1.39)	1.22*** (1.07–1.39)	1.13** (1.01–1.26)	1.10 (0.97–1.24)
Non-black African (vs black African)	0.40* (0.14–1.14)	0.03*** (0.01–0.08)	0.11*** (0.05–0.27)	0.07*** (0.03–0.18)	0.10*** (0.03–0.27)
Married (vs unmarried)	0.75 (0.21–2.74)	0.33** (0.14–0.80)	0.32** (0.12–0.87)	0.46 (0.14–1.47)	
Marital status (ref = single)					
Married					0.72 (0.21–2.44)
Cohabitating	–	–	–	–	2.12** (1.08–4.17)
Locality (ref = urban formal)					
Urban informal	1.25 (0.47–3.31)	1.78* (0.98–3.26)	1.45 (0.78–2.72)	1.27 (0.68–2.39)	1.28 (0.64–2.57)
Rural informal	0.41* (0.15–1.08)	0.87 (0.50–1.49)	0.97 (0.53–1.77)	1.27 (0.76–2.11)	1.54 (0.91–2.60)
Rural formal	1.35 (0.37–4.89)	1.03 (0.46–2.30)	1.69 (0.80–3.55)	1.54 (0.80–2.99)	1.51 (0.76–2.97)
Employment status (ref = unemployed)					
Student	0.41 (0.13–1.23)	1.45 (0.73–2.86)	0.61 (0.30–1.25)	0.46** (0.23–0.94)	0.40*** (0.21–0.78)
Employed	0.93 (0.37–2.35)	0.97 (0.42–2.25)	0.91 (0.40–2.04)	0.79 (0.41–1.51)	1.17 (0.54–2.53)
Condom used at last sex (vs not used)	1.09 (0.47–2.53)	0.97 (0.56–1.67)	1.28 (0.74–2.21)	2.11*** (1.36–3.27)	2.69*** (1.72–4.22)
Age of first sex ≤15 (vs aged 16+)	1.06 (0.41–2.71)	1.16 (0.65–2.06)	1.45 (0.80–2.61)	1.49 (0.83–2.67)	1.55 (0.78–3.05)
Multiple sexual partners in past year (vs 1 partner)	1.78 (0.63–5.02)	2.81*** (1.32–5.96)	1.41 (0.56–3.53)	1.64 (0.83–3.23)	–
Number of lifetime partners (ref = 1)					
2 lifetime partners	–	–	–	–	0.87 (0.46–1.63)
3–4 lifetime partners	–	–	–	–	1.61 (0.85–3.03)
5+ lifetime partners	–	–	–	–	1.09 (0.41–2.91)
Think partner had other partners (vs did not think)	–	–	–	–	1.95** (1.16–3.27)
Income last month >R4000 (vs ≤R4000)	–	–	–	–	0.06*** (0.01–0.41)

Note: *=p<0.10; **=p<0.05; and ***=p<0.01

All analyses were adjusted to account for the complex study design (i.e. stratification and clustering) and for non-response (by using weighted data).

**Table S4.** Multiple regression analyses showing associations between years age difference between partners (continuous) and covariates with HIV status for young women aged 15 to 24 years, South Africa 2002, 2005, 2008, and 2012.

	2002 (A) N=446	2005 (A) N=994	2008 (A) N=854	2012 (A) N=1394	2012 (B) N=1257
	aOR (95% CI)	aOR (95% CI)	aOR (95% CI)	aOR (95% CI)	aOR (95% CI)
Years age difference (continuous)	1.10 (0.98–1.22)	1.10*** (1.03–1.17)	1.08** (1.01–1.15)	1.08** (1.01–1.16)	1.08** (1.01–1.15)
Age	1.04 (0.87–1.25)	1.22*** (1.08–1.37)	1.21*** (1.06–1.38)	1.13** (1.01–1.26)	1.10 (0.98–1.24)
Non-black African (vs black African)	0.37* (0.11–1.19)	0.03*** (0.01–0.08)	0.11*** (0.05–0.27)	0.07*** (0.03–0.18)	0.10*** (0.03–0.28)
Married (vs unmarried)	0.70 (0.22–2.28)	0.33** (0.14–0.81)	0.31** (0.11–0.85)	0.41 (0.13–1.34)	
Marital status (ref = single)					
Married					0.68 (0.20–2.29)
Cohabitating	–	–	–	–	1.88* (0.95–3.71)
Locality (ref = urban formal)					
Urban informal	1.20 (0.45–3.21)	1.65 (0.90–3.01)	1.49 (0.80–2.75)	1.22 (0.64–2.31)	1.23 (0.61–2.48)
Rural informal	0.42* (0.16–1.12)	0.79 (0.45–1.39)	0.98 (0.53–1.79)	1.23 (0.74–2.04)	1.47 (0.87–2.47)
Rural formal	1.35 (0.37–4.91)	0.95 (0.43–2.12)	1.65 (0.80–3.40)	1.51 (0.78–2.93)	1.48 (0.75–2.94)
Employment status (ref = unemployed)					
Student	0.40 (0.13–1.23)	1.44 (0.73–2.87)	0.59 (0.29–1.20)	0.48** (0.24–0.99)	0.41*** (0.21–0.79)
Employed	0.94 (0.36–2.42)	0.93 (0.39–2.22)	0.93 (0.42–2.08)	0.80 (0.41–1.55)	1.14 (0.52–2.47)
Condom used at last sex (vs not used)	1.12 (0.49–2.55)	0.98 (0.57–1.68)	1.24 (0.70–2.17)	2.18*** (1.42–3.35)	2.65*** (1.69–4.16)
Age of first sex ≤15 (vs 16+)	1.03 (0.37–2.84)	1.15 (0.66–2.01)	1.44 (0.80–2.59)	1.45 (0.80–2.61)	1.52 (0.78–2.98)
Multiple sexual partners in past year (vs 1 partner)	1.72 (0.62–4.78)	2.77*** (1.29–5.93)	1.43 (0.59–3.45)	1.50 (0.77–2.94)	–
Number of lifetime partners (ref = 1)					
2 lifetime partners	–	–	–	–	0.85 (0.45–1.61)
3–4 lifetime partners	–	–	–	–	1.55 (0.82–2.91)
5+ lifetime partners	–	–	–	–	1.07 (0.41–2.77)
Think partner had other partners (vs did not think)	–	–	–	–	1.96** (1.17–3.27)
Income last month >R4000 (vs ≤R4000)	–	–	–	–	0.07*** (0.01–0.43)

Note: *=p<0.10; **=p<0.05; and ***=p<0.01

All analyses were adjusted to account for the complex study design (i.e. stratification and clustering) and for non-response (by using weighted data).

**Table S5.** Bivariate regression analyses showing associations between age-disparate sex (5+ years older) and years age difference between partners (continuous) considering the most recent partner only and HIV status for young women aged 15–24 years, South Africa 2002, 2005, 2008, and 2012.

	2002 OR (95% CI) N=495	2005 OR (95% CI) N=1063	2008 OR (95% CI) N=876	2012 OR (95% CI) N=1490
Age-disparate sex 5+ yrs older (vs ≤4yrs)	1.73 (0.86–3.45)	2.16*** (1.35–3.46)	1.90*** (1.17–3.07)	1.26 (0.78–2.03)
Years age difference (continuous)	1.07 (0.98–1.18)	1.10*** (1.04–1.16)	1.07** (1.00–1.14)	1.08** (1.02–1.15)

Note: *=p<0.10; **=p<0.05; and ***=p<0.01

All analyses were adjusted to account for the complex study design (i.e. stratification and clustering) and for non-response (by using weighted data).

**Table S6.** Multiple regression analyses showing associations between age-disparate sex (5+ years older, considering most recent partner only) and covariates with HIV status for young women aged 15 to 24 years, South Africa 2002, 2005, 2008, and 2012.

	2002 (A) N=446	2005 (A) N=994	2008 (A) N=854	2012 (A) N=1394	2012 (B) N=1257
	aOR (95% CI)	aOR (95% CI)	aOR (95% CI)	aOR (95% CI)	aOR (95% CI)
Age-disparate sex 5+ yrs older (vs ≤4yrs)	1.74 (0.81–3.77)	2.09*** (1.22–3.59)	2.01*** (1.23–3.29)	1.29 (0.78–2.12)	1.54* (0.93–2.55)
Age	1.04 (0.86–1.25)	1.23*** (1.09–1.39)	1.22*** (1.07–1.39)	1.13** (1.01–1.26)	1.20 (0.97–1.24)
Non-black African (vs black African)	0.40* (0.14–1.14)	0.03*** (0.01–0.08)	0.11*** (0.05–0.27)	0.07*** (0.03–0.18)	0.10*** (0.03–0.27)
Married (vs unmarried)	0.75 (0.21–2.74)	0.33** (0.14–0.81)	0.32** (0.12–0.87)	0.46 (0.14–1.47)	
Marital status (ref = single)					
Married					0.71 (0.21–2.44)
Cohabitating	–				2.12** (1.08–4.17)
Locality (ref = urban formal)					
Urban informal	1.25 (0.47–3.31)	1.76* (0.96–3.22)	1.46 (0.78–2.73)	1.27 (0.68–2.39)	1.28 (0.64–2.56)
Rural informal	0.41 (0.13–1.23)	0.86 (0.50–1.49)	0.97 (0.53–1.77)	1.27 (0.76–2.11)	1.54 (0.91–2.60)
Rural formal	1.34 (0.37–4.89)	1.02 (0.46–2.30)	1.68 (0.80–3.54)	1.54 (0.80–3.00)	1.50 (0.76–2.97)
Employment status (ref = unemployed)					
Student	0.40 (0.13–1.23)	1.48 (0.74–2.94)	0.61 (0.30–1.24)	0.46** (0.23–0.94)	0.40*** (0.21–0.78)
Employed	0.93 (0.37–2.35)	0.97 (0.42–2.24)	0.90 (0.40–2.04)	0.79 (0.41–1.51)	1.17 (0.54–2.53)
Condom used at last sex (vs not used)	1.09 (0.47–2.52)	0.96 (0.56–1.65)	1.28 (0.74–2.22)	2.11*** (1.36–3.27)	2.70*** (1.72–4.22)
Age of first sex ≤15 (vs aged 16+)	1.05 (0.41–2.71)	1.14 (0.64–2.04)	1.45 (0.80–2.62)	1.48 (0.83–2.66)	1.55 (0.78–3.05)
Multiple sexual partners in past year (vs 1 partner)	1.78 (0.63–5.04)	3.33*** (1.60–6.95)	–1.44 (0.57–3.63)	1.64 (0.83–3.24)	–
Number of lifetime partners (ref = 1)					
2 lifetime partners	–	–	–	–	0.87 (0.46–1.63)
3–4 lifetime partners	–	–	–	–	1.61 (0.85–3.04)
5+ lifetime partners	–	–	–	–	1.09 (0.41–2.91)
Think partner had other partners (vs did not think)	–	–	–	–	1.95** (1.17–3.27)
Income last month >R4000 (vs ≤R4000)	–	–	–	–	0.06*** (0.01–0.41)

Note: *=p<0.10; **=p<0.05; and ***=p<0.01

All analyses were adjusted to account for the complex study design (i.e. stratification and clustering) and for non-response (by using weighted data).

**Table S7.** Multiple regression analyses showing associations between years age difference between partners (most recent partner only) and covariates with HIV status for young women aged 15–24 years, South Africa 2002, 2005, 2008, and 2012.

	2002 (A) N=446	2005 (A) N=994	2008 (A) N=854	2012 (A) N=1394	2012 (B) N=1257
	aOR (95% CI)	aOR (95% CI)	aOR (95% CI)	aOR (95% CI)	aOR (95% CI)
Years age difference (continuous)	1.09 (0.97–1.22)	1.10*** (1.03–1.17)	1.07** (1.00–1.15)	1.09** (1.02–1.16)	1.08** (1.01–1.15)
Age	1.04 (0.87–1.25)	1.22*** (1.08–1.37)	1.21*** (1.06–1.37)	1.12** (1.01–1.25)	1.10 (0.97–1.24)
Non-black African (vs black African)	0.37* (0.12–1.18)	0.03*** (0.01–0.08)	0.11*** (0.04–0.27)	0.07*** (0.03–0.18)	0.10*** (0.03–0.28)
Married (vs unmarried)	0.71 (0.22–2.30)	0.33** (0.13–0.80)	0.32** (0.12–0.86)	0.41 (0.12–1.34)	
Marital status (ref = single)					
Married					0.67 (0.20–2.28)
Cohabitating	–				1.87* (0.94–3.69)
Locality (ref = urban formal)					
Urban informal	1.22 (0.46–3.24)	1.64 (0.89–3.00)	1.51 (0.81–2.78)	1.22 (0.64–2.31)	1.23 (0.61–2.49)
Rural informal	0.42* (0.16–1.12)	0.80 (0.46–1.40)	0.98 (0.53–1.79)	1.22 (0.74–2.03)	1.46 (0.86–2.47)
Rural formal	1.36 (0.37–4.90)	0.94 (0.42–2.11)	1.65 (0.80–3.41)	1.51 (0.78–2.93)	1.48 (0.75–2.93)
Employment status (ref = unemployed)					
Student	0.40 (0.13–1.23)	1.46 (0.74–2.91)	0.59 (0.42–2.08)	0.48** (0.24–0.99)	0.41*** (0.21–0.79)
Employed	0.93 (0.36–2.41)	0.94 (0.40–2.24)	0.93 (0.42–2.08)	0.80 (0.41–1.55)	1.13 (0.52–2.46)
Condom used at last sex (vs not used)	1.11 (0.48–2.53)	0.98 (0.57–1.68)	1.23 (0.70–2.16)	2.19*** (1.43–3.37)	2.67*** (1.70–4.18)
Age of first sex ≤15 (vs 16+)	1.03 (0.38–2.84)	1.14 (0.65–1.99)	1.44 (0.80–2.58)	1.55 (0.80–3.02)	1.51 (0.77–2.96)
Multiple sexual partners in past year (vs 1 partner)	1.76 (0.62–4.95)	3.44*** (1.64–7.23(	1.48 (0.60–3.65)	1.44 (0.80–2.60)	–
Number of lifetime partners (ref = 1 partner)					
2 lifetime partners	–	–	–	–	0.85 (0.45–1.61)
3–4 lifetime partners	–	–	–	–	1.55 (0.82–2.92)
5+ lifetime partners	–	–	–	–	1.09 (0.42–2.81)
Think partner had other partners (vs did not think)	–	–	–	–	1.96** (1.17–3.28)
Income last month >R4000 (vs ≤R4000)	–	–	–	–	0.06*** (0.01–0.43)

Note: *=p<0.10; **=p<0.05; and ***=p<0.01

All analyses were adjusted to account for the complex study design (i.e. stratification and clustering) and for non-response (by using weighted data).

**Table S8.** Bivariate regression analyses showing associations between age-disparate sex (5+ years older) and years age difference between partners (continuous) and HIV status for young black African women aged 15 to 24 years, South Africa 2002, 2005, 2008, and 2012.

	2002 OR (95% CI) N=350	2005 OR (95% CI) N=803	2008 OR (95% CI) N=661	2012 OR (95% CI) N=1116
Age-disparate sex 5+yrs older (vs age similar ≤4yrs)	2.03* (0.96–4.26)	2.30*** (1.39–3.80)	1.88** (1.17–3.03)	1.22 (0.74–2.01)
Years age difference between partners (continuous)	1.13** (1.02–1.26)	1.10*** (1.04–1.17)	1.07** (1.00–1.15)	1.08** (1.01–1.15)

Note: *=p<0.10; **=p<0.05; and ***=p<0.01

All analyses were adjusted to account for the complex study design (i.e. stratification and clustering) and for non-response (by using weighted data).

**Table S9.** Multiple regression analyses showing associations between age-disparate sex (5+ years older) and covariates with HIV status for young black African women aged 15–24 years, South Africa 2002, 2005, 2008, and 2012.

	2002 (A) N=309	2005 (A) N=746	2008 (A) N=642	2012 (A) N=1039	2012 (B) N=939
	aOR (95% CI)	aOR (95% CI)	aOR (95% CI)	aOR (95% CI)	aOR (95% CI)
Years age difference (continuous)	2.39** (1.01–5.64)	2.11*** (1.21–3.68)	1.97*** (1.20–3.24)	1.24 (0.75–2.06)	1.48 (0.88–2.46)
Age	1.13 (0.91–1.40)	1.24*** (1.09–1.40)	1.22***(1.06–1.39)	1.12** (1.00–1.25)	1.09 (0.96–1.23)
Married (vs unmarried)	0.77 (0.19–3.14)	0.32** (0.13–0.80)	0.33** (0.12–0.91)	0.47 (0.14–1.53)	
Marital status (ref = single)					
Married					0.74 (0.22–2.54)
Cohabitating					2.22** (1.11–4.44)
Locality (ref = urban formal)					
Urban informal	1.06 (0.38–2.89)	1.79* (0.97–3.28)	1.42 (0.75–2.69)	1.28 (0.68–2.41)	1.31 (0.65–2.66)
Rural informal	0.35** (0.13–0.92)	0.87 (0.50–1.50)	0.96 (0.52–1.76)	1.27 (0.76–2.11)	1.55 (0.92–2.62)
Rural formal	0.80 (0.21–3.03)	1.03 (0.46–2.33)	1.64 (0.77–3.52)	1.48 (0.73–2.98)	1.55 (0.76–3.15)
Employment status (ref = unemployed)					
Student	0.47 (0.15–1.46)	1.47 (0.74–2.90)	0.60 (0.29–1.25)	0.46** (0.22–0.94)	0.40*** (0.20–0.78)
Employed	0.43 (0.15–1.28)	0.96 (0.40–2.29)	0.91 (0.38–2.16)	0.82 (0.42–1.57)	1.22 (0.56–2.68)
Condom used at last sex (vs not used)	1.03* (0.41–2.58)	0.97 (0.56–1.68)	1.24 (0.71–2.18)	2.06*** (1.32–3.21)	2.62*** (1.66–4.12)
Age of first sex ≤15 (vs 16+)	0.83 (0.31–2.21)	1.18 (0.66–2.11)	1.45 (0.79–2.66)	1.46 (0.81–2.66)	1.52 (0.75–3.05)
Multiple sexual partners in past year (vs 1 partner)	2.64* (0.84–8.26)	2.95*** (1.36–6.40)	1.49 (0.58–3.82)	1.66 (0.84–3.30)	
Number of lifetime partners (ref = 1 partner)					
2 lifetime partners	–	–	–	–	0.82 (0.43–1.57)
3–4 lifetime partners	–	–	–	–	1.61 (0.85–3.07)
5+ lifetime partners	–	–	–	–	1.10 (0.41–3.00)
Think partner had MSPs (vs did not think)	–	–	–	–	1.92** (1.14–3.23)
Income last month >R4000 (vs ≤R4000)	–	–	–	–	0.06*** (0.01–0.45)

Note: *=p<0.10; **=p<0.05; and ***=p<0.01

All analyses were adjusted to account for the complex study design (i.e. stratification and clustering) and for non-response (by using weighted data).

**Table S10.** Multiple regression analyses showing associations between years age difference between partners (continuous) and covariates with HIV status for young black women aged 15 to 24 years, South Africa 2002, 2005, 2008, and 2012.

	2002 (A) N=309	2005 (A) N=994	2008 (A) N=642	2012 (A) N=1039	2012 (B) N=939
	aOR (95% CI)	aOR (95% CI)	aOR (95% CI)	aOR (95% CI)	aOR (95% CI)
Years age difference (continuous)	1.21*** (1.07–1.37)	1.09*** (1.03–1.16)	1.08** (1.01–1.16)	1.08** (1.01–1.16)	1.08** (1.01–1.15)
Age	1.14 (0.92–1.40)	1.22*** (1.08–1.38)	1.21*** (1.05–1.38)	1.12** (1.00–1.25)	1.09 (0.96–1.23)
Married (vs unmarried)	0.55 (0.14–2.13)	0.33** (0.13–0.81)	0.32** (0.12–0.89)	0.42 (0.13–1.39)	0.70 (0.20–2.37)
Marital status (ref = single)					
Married					
Cohabitating	–				
Locality (ref = urban formal)					
Urban informal	0.94 (0.33–2.73)	1.65 (0.90–3.03)	1.45 (0.77–2.73)	1.23 (0.64–2.34)	1.26 (0.62–2.57)
Rural informal	0.35** (0.13–0.95)	0.79 (0.45–1.40)	0.97 (0.53–1.78)	1.23 (0.74–2.04)	1.48 (0.88–2.50)
Rural formal	0.70 (0.20–2.52)	0.95 (0.42–2.14)	1.60 (0.76–3.38)	1.46 (0.72–2.95)	1.55 (0.76–3.16)
Employment status (ref = unemployed)					
Student	0.45 (0.14–1.42)	1.46 (0.73–2.89)	0.58 (0.28–1.21)	0.48** (0.23–0.99)	0.40*** (0.21–0.78)
Employed	0.38* (0.13–1.10)	0.92 (0.37–2.26)	0.93 (0.40–2.18)	0.82 (0.42–1.61)	1.18 (0.53–2.60)
Condom used at last sex (vs not used)	1.01** (0.41–2.48)	0.98 (0.57–1.69)	1.20 (0.67–2.14)	2.13*** (1.38–3.29)	2.58*** (1.63–4.07)
Age of first sex ≤15 (vs 16+)	0.65 (0.21–2.02)	1.17 (0.67–2.06)	1.45 (0.79–2.64)	1.43 (0.78–2.60)	1.49 (0.75–2.98)
Multiple sexual partners in past year (vs 1 partner)	2.71* (0.90–8.17)	2.91*** (1.33–6.37)	1.50 (0.60–3.73)	1.52 (0.77–3.00)	
Number of lifetime partners (ref = 1)					
2 lifetime partners	–	–	–	–	0.80 (0.42–1.54)
3–4 lifetime partners	–	–	–	–	1.55 (0.82–2.96)
5+ lifetime partners	–	–	–	–	1.08 (0.41–2.86)
Think partner had MSPs (vs did not think)	–	–	–	–	1.93** (1.15–3.24)
Income last month >R4000 (vs ≤R4000)	–	–	–	–	0.07*** (0.01–0.48)

Note: *=p<0.10; **=p<0.05; and ***=p<0.01

All analyses were adjusted to account for the complex study design (i.e. stratification and clustering) and for non-response (by using weighted data).

## Additional file 1

**Supplementary Digital Content**

Sexual behaviour data, bivariate regression analyses, full models of the multiple regression analyses, and sensitivity analyses (restricted to most recent partner only and black African women only).

**Supplementary Digital Content.pdf**
