# Supplementary material for: Age-disparate sex and HIV risk for young women from 2002 to 2012 in South Africa
Source: J Int AIDS Soc. 2016 Dec 26;19(1):21310. doi: 10.7448/IAS.19.1.21310 (PMC5384594; doi:10.7448/IAS.19.1.21310)
Supplement: Supplementary material [file zias_a_1268794_sm5159.docx]

**Age-disparate sex and HIV risk for young women from 2002 to 2012 in South Africa**

**Supplementary Digital Content**

Meredith GB Evans; Kathryn Risher; Nompumelelo Zungu; Olive Shisana; Sizulu Moyo; David D Celentano; Brendan Maughan-Brown; Thomas M Rehle

**Table S1.** Sexual behaviour reported by young women aged 15 to 24 years, South Africa 2002, 2005, 2008, and 2012.

|  | **2002**  **n (%)** | **2005**  **n (%)** | **2008**  **n (%)** | **2012**  **n (%)** |
| --- | --- | --- | --- | --- |
| **Condom use at last sex** | N = 550 | N = 1072 | N = 897 | N = 1474 |
| Yes | 250 (45.4) | 530 (53.0) | 546 (62.7) | 772 (50.6) |
| No or do not know | 300 (54.6) | 542 (47.0) | 351 (37.3) | 702 (49.4) |
| **Consistent condom use with most recent partner*** | | | N = 886 | N = 1480 |
| Every time | - | - | 428 (50.7) | 558 (36.9) |
| Almost every time | - | - | 55 (5.0) | 70 (6.9) |
| Sometimes | - | - | 205 (24.1) | 313 (22.0) |
| Never | - | - | 198 (20.1) | 539 (34.1) |
| **Age at first sex** | N = 544 | N = 1044 | N = 890 | N = 1776 |
| Aged ≤15 | 112 (20.7) | 186 (15.5) | 156 (18.5) | 305 (15.5) |
| Aged 16+ | 432 (79.3) | 858 (84.5) | 734 (81.5) | 1471 (84.5) |
| **Multiple sexual partnerships** | N = 557 | N = 1063 | N = 903 | N = 1497 |
| 1 partner | 507 (91.4) | 1002 (94.1) | 840 (93.5) | 1376 (91.7) |
| 2+ partners | 50 (8.6) | 61 (5.9) | 63 (6.5) | 121 (8.3) |
| **Number of lifetime partners** | |  |  | N = 1446 |
| 1 lifetime partner | - | - | - | 624 (43.5) |
| 2 lifetime partners | - | - | - | 369 (25.7) |
| 3-4 lifetime partners | - | - | - | 326 (23.2) |
| 5+ lifetime partners | - | - | - | 127 (7.5) |
| **Think partner had other sexual partners in past year** | | |  | N = 1454 |
| Yes or do not know | - | - | - | 738 (55.1) |
| No | - | - | - | 716 (44.9) |

*Consistent condom use was not measured in 2002 or 2005. Condom use at last sex was therefore included in the multiple regression analyses because it was measured in all survey years and because the measure of condom use at last sex is less subject to recall bias than consistenct condom use.

| **Table S2.** Bivariate regression analyses showing associations between select sociodemographic and sexual behaviours with HIV status for young women aged 15 to 24 years, South Africa 2002, 2005, 2008, and 2012. | | | | |
| --- | --- | --- | --- | --- |
|  | **2002 OR (95% CI)** | **2005 OR (95% CI)** | **2008 OR (95% CI)** | **2012 OR (95% CI)** |
| Age-disparate sex 5+yrs older (vs ≤4yrs) | 1.73 (0.86-3.45) | 2.28*** (1.41-3.69) | 1.88*** (1.17-3.03) | 1.25 (0.77-2.02) |
| Age difference in years (continuous) | 1.08 (0.98-1.19) | 1.10*** (1.04-1.16) | 1.07** (1.00-1.14) | 1.08** (1.02-1.15) |
| *Sociodemographics* |  |  |  |  |
| Age | 1.09 (0.94-1.26) | 1.19*** (1.07-1.32) | 1.20*** (1.08-1.33) | 1.15*** (1.05-1.25) |
| Non-black African (vs black African) | 0.56 (0.19-1.65) | 0.05*** (0.02-0.15) | 0.12*** (0.05-0.29) | 0.06*** (0.02-0.15) |
| Married (vs unmarried) | 0.81 (0.28-2.34) |  |  |  |
| Marital status (ref = single) |  |  |  |  |
| Married |  | 0.32*** (0.15-0.67) | 0.47* (0.19-1.14) | 0.43 (0.14-1.29) |
| Cohabitating |  | 0.74 (0.33-1.65) | 1.29 (0.59-2.80) | 1.31 (0.70-2.46) |
| Locality (ref = urban formal) |  |  |  |  |
| Urban informal | 2.13* (0.87-5.18) | 2.37*** (1.38-4.08) | 1.93** (1.09-3.41) | 1.81* (0.92-3.56) |
| Rural informal | 0.52 (0.23-1.18) | 1.10 (0.65-1.85) | 1.02 (0.55-1.86) | 1.56* (0.98-2.48) |
| Rural formal | 1.5 (0.52-4.36) | 1.07 (0.53-2.16) | 1.90* (0.91-3.96) | 0.79 (0.38-1.64) |
| Employment status (ref = unemployed) | |  |  |  |
| Student | 0.45* (0.18-1.13) | 0.88 (0.48-1.63) | 0.47** (0.25-0.88) | 0.46*** (0.26-0.81) |
| Employed | 0.94 (0.36-2.45) | 0.63 (0.33-1.21) | 0.8 (0.40-1.60) | 0.64 (0.34-1.20) |
| *Sexual Behaviour* |  |  |  |  |
| Condom used at last sex (vs condom not used) | 1.23 (0.62-2.45) | 1.17 (0.76-1.82) | 1.09 (0.66-1.79) | 1.85*** (1.21-2.83) |
| Aged ≤15 at first sex (vs aged 16+) | 0.87 (0.39-1.95) | 0.98 (0.58-1.68) | 1.13 (0.65-1.97) | 1.18 (0.65-2.15) |
| Multiple sexual partners in past year (vs 1 partner) | 1.97 (0.76-5.07) | 2.74*** (1.36-5.54) | 1.03 (0.43-2.44) | 1.45 (0.78-2.72) |
| *Additional variables measured in 2012* | |  |  |  |
| Income past month >R4000 (vs ≤R4000) | - | - | - | 0.13*** (0.04-0.44) |
| Number of lifetime partners (ref = 1 partner) | |  |  |  |
| 2 lifetime partners | - | - | - | 1.08 (0.61-1.91) |
| 3-4 lifetime partners | - | - | - | 2.15*** (1.22-3.78) |
| 5+ lifetime partners | - | - | - | 1.43 (0.62-3.26) |
| Think partner had other sexual partners (vs did not think) | - | - | - | 2.48*** (1.54-3.99) |

Note: *=p<0.10; **=p<0.05; and ***=p<0.01

All analyses were adjusted to account for the complex study design (i.e. stratification and clustering) and for survey non-response (by using weighted data).

**Full regression models showing associations between age-disparate sex and covariates with HIV status and between age difference with partner and covariates with HIV status for young women aged 15 to 24 years, South Africa 2002, 2005, 2008, and 2012.**

**Table S3.** Multiple regression analyses showing associations between age-disparate sex (5+ years older) and covariates with HIV status for young women aged 15 to 24 years, South Africa 2002, 2005, 2008, and 2012.

|  | **2002 (A)**  **N=446** | **2005 (A)**  **N=994** | **2008 (A)**  **N=854** | **2012 (A)**  **N=1394** | **2012 (B)**  **N=1257** |
| --- | --- | --- | --- | --- | --- |
|  | **aOR (95% CI)** | **aOR (95% CI)** | **aOR (95% CI)** | **aOR (95% CI)** | **aOR (95% CI)** |
| Age-disparate 5+yrs older (vs ≤4yrs) | 1.74 (0.81-3.76) | 2.11*** (1.22-3.66) | 2.02*** (1.24-3.29) | 1.28 (0.78-2.10) | 1.53* (0.92-2.54) |
| Age | 1.04 (0.86-1.25) | 1.23*** (1.09-1.39) | 1.22*** (1.07-1.39) | 1.13** (1.01-1.26) | 1.10 (0.97-1.24) |
| Non-black African (vs black African) | 0.40* (0.14-1.14) | 0.03*** (0.01-0.08) | 0.11*** (0.05-0.27) | 0.07*** (0.03-0.18) | 0.10*** (0.03-0.27) |
| Married (vs unmarried) | 0.75 (0.21-2.74) | 0.33** (0.14-0.80) | 0.32** (0.12-0.87) | 0.46 (0.14-1.47) |  |
| Marital status (ref = single) | |  |  |  |  |
| Married |  |  |  |  | 0.72 (0.21-2.44) |
| Cohabitating | - | - | - | - | 2.12** (1.08-4.17) |
| Locality (ref = urban formal) | |  |  |  |  |
| Urban informal | 1.25 (0.47-3.31) | 1.78* (0.98-3.26) | 1.45 (0.78-2.72) | 1.27 (0.68-2.39) | 1.28 (0.64-2.57) |
| Rural informal | 0.41* (0.15-1.08) | 0.87 (0.50-1.49) | 0.97 (0.53-1.77) | 1.27 (0.76-2.11) | 1.54 (0.91-2.60) |
| Rural formal | 1.35 (0.37-4.89) | 1.03 (0.46-2.30) | 1.69 (0.80-3.55) | 1.54 (0.80-2.99) | 1.51 (0.76-2.97) |
| Employment status (ref = unemployed) |  |  |  |  |  |
| Student | 0.41 (0.13-1.23) | 1.45 (0.73-2.86) | 0.61 (0.30-1.25) | 0.46** (0.23-0.94) | 0.40*** (0.21-0.78) |
| Employed | 0.93 (0.37-2.35) | 0.97 (0.42-2.25) | 0.91 (0.40-2.04) | 0.79 (0.41-1.51) | 1.17 (0.54-2.53) |
| Condom used at last sex (vs not used) | 1.09 (0.47-2.53) | 0.97 (0.56-1.67) | 1.28 (0.74-2.21) | 2.11*** (1.36-3.27) | 2.69*** (1.72-4.22) |
| Age of first sex ≤15 (vs aged 16+) | 1.06 (0.41-2.71) | 1.16 (0.65-2.06) | 1.45 (0.80-2.61) | 1.49 (0.83-2.67) | 1.55 (0.78-3.05) |
| Multiple sexual partners in past year (vs 1 partner) | 1.78 (0.63-5.02) | 2.81*** (1.32-5.96) | 1.41 (0.56-3.53) | 1.64 (0.83-3.23) | - |
| Number of lifetime partners (ref = 1) | |  |  |  |  |
| 2 lifetime partners | - | - | - | - | 0.87 (0.46-1.63) |
| 3-4 lifetime partners | - | - | - | - | 1.61 (0.85-3.03) |
| 5+ lifetime partners | - | - | - | - | 1.09 (0.41-2.91) |
| Think partner had other partners (vs did not think) | - | - | - | - | 1.95** (1.16-3.27) |
| Income last month >R4000 (vs ≤R4000) | - | - | - | - | 0.06*** (0.01-0.41) |

Note: *=p<0.10; **=p<0.05; and ***=p<0.01

All analyses were adjusted to account for the complex study design (i.e. stratification and clustering) and for non-response (by using weighted data).

**Table S4.** Multiple regression analyses showing associations between years age difference between partners (continuous) and covariates with HIV status for young women aged 15 to 24 years, South Africa 2002, 2005, 2008, and 2012.

|  | **2002 (A)**  **N=446** | **2005 (A)**  **N=994** | **2008 (A)**  **N=854** | **2012 (A)**  **N=1394** | **2012 (B)**  **N=1257** |
| --- | --- | --- | --- | --- | --- |
|  | **aOR (95% CI)** | **aOR (95% CI)** | **aOR (95% CI)** | **aOR (95% CI)** | **aOR (95% CI)** |
| Years age difference (continuous) | 1.10 (0.98-1.22) | 1.10*** (1.03-1.17) | 1.08** (1.01-1.15) | 1.08** (1.01-1.16) | 1.08** (1.01-1.15) |
| Age | 1.04 (0.87-1.25) | 1.22*** (1.08-1.37) | 1.21*** (1.06-1.38) | 1.13** (1.01-1.26) | 1.10 (0.98-1.24) |
| Non-black African (vs black African) | 0.37* (0.11-1.19) | 0.03*** (0.01-0.08) | 0.11*** (0.05-0.27) | 0.07*** (0.03-0.18) | 0.10*** (0.03-0.28) |
| Married (vs unmarried) | 0.70 (0.22-2.28) | 0.33** (0.14-0.81) | 0.31** (0.11-0.85) | 0.41 (0.13-1.34) |  |
| Marital status (ref = single) |  |  |  |  |  |
| Married |  |  |  |  | 0.68 (0.20-2.29) |
| Cohabitating | - | - | - | - | 1.88* (0.95-3.71) |
| Locality (ref = urban formal) |  |  |  |  |  |
| Urban informal | 1.20 (0.45-3.21) | 1.65 (0.90-3.01) | 1.49 (0.80-2.75) | 1.22 (0.64-2.31) | 1.23 (0.61-2.48) |
| Rural informal | 0.42* (0.16-1.12) | 0.79 (0.45-1.39) | 0.98 (0.53-1.79) | 1.23 (0.74-2.04) | 1.47 (0.87-2.47) |
| Rural formal | 1.35 (0.37-4.91) | 0.95 (0.43-2.12) | 1.65 (0.80-3.40) | 1.51 (0.78-2.93) | 1.48 (0.75-2.94) |
| Employment status (ref = unemployed) | |  |  |  |  |
| Student | 0.40 (0.13-1.23) | 1.44 (0.73-2.87) | 0.59 (0.29-1.20) | 0.48** (0.24-0.99) | 0.41*** (0.21-0.79) |
| Employed | 0.94 (0.36-2.42) | 0.93 (0.39-2.22) | 0.93 (0.42-2.08) | 0.80 (0.41-1.55) | 1.14 (0.52-2.47) |
| Condom used at last sex (vs not used) | 1.12 (0.49-2.55) | 0.98 (0.57-1.68) | 1.24 (0.70-2.17) | 2.18*** (1.42-3.35) | 2.65*** (1.69-4.16) |
| Age of first sex ≤15 (vs 16+) | 1.03 (0.37-2.84) | 1.15 (0.66-2.01) | 1.44 (0.80-2.59) | 1.45 (0.80-2.61) | 1.52 (0.78-2.98) |
| Multiple sexual partners in past year (vs 1 partner) | 1.72 (0.62-4.78) | 2.77*** (1.29-5.93) | 1.43 (0.59-3.45) | 1.50 (0.77-2.94) | - |
| Number of lifetime partners (ref = 1) | |  |  |  |  |
| 2 lifetime partners | - | - | - | - | 0.85 (0.45-1.61) |
| 3-4 lifetime partners | - | - | - | - | 1.55 (0.82-2.91) |
| 5+ lifetime partners | - | - | - | - | 1.07 (0.41-2.77) |
| Think partner had other partners (vs did not think) | - | - | - | - | 1.96** (1.17-3.27) |
| Income last month >R4000 (vs ≤R4000) | - | - | - | - | 0.07*** (0.01-0.43) |

Note: *=p<0.10; **=p<0.05; and ***=p<0.01

All analyses were adjusted to account for the complex study design (i.e. stratification and clustering) and for non-response (by using weighted data).

**Sensitivity analysis considering young women’s most recent partner only**

**Table S5.** Bivariate regression analyses showing associations between age-disparate sex (5+ years older) and years age difference between partners (continuous) considering the most recent partner only and HIV status for young women aged 15 to 24 years, South Africa 2002, 2005, 2008, and 2012.

|  | **2002 OR (95% CI)**  **N=495** | **2005 OR (95% CI)**  **N=1063** | **2008 OR (95% CI)**  **N=876** | **2012 OR (95% CI)**  **N=1490** |
| --- | --- | --- | --- | --- |
| Age-disparate sex 5+yrs older (vs ≤4yrs) | 1.73 (0.86-3.45) | 2.16*** (1.35-3.46) | 1.90*** (1.17-3.07) | 1.26 (0.78-2.03) |
| Years age difference (continuous) | 1.07 (0.98-1.18) | 1.10*** (1.04-1.16) | 1.07** (1.00-1.14) | 1.08** (1.02-1.15) |

Note: *=p<0.10; **=p<0.05; and ***=p<0.01

All analyses were adjusted to account for the complex study design (i.e. stratification and clustering) and for non-response (by using weighted data).

**Table S6.** Multiple regression analyses showing associations between age-disparate sex (5+ years older, considering most recent partner only) and covariates with HIV status for young women aged 15 to 24 years, South Africa 2002, 2005, 2008, and 2012.

|  | **2002 (A)**  **N=446** | **2005 (A)**  **N=994** | **2008 (A)**  **N=854** | **2012 (A)**  **N=1394** | **2012 (B)**  **N=1257** |
| --- | --- | --- | --- | --- | --- |
|  | **aOR (95% CI)** | **aOR (95% CI)** | **aOR (95% CI)** | **aOR (95% CI)** | **aOR (95% CI)** |
| Age-disparate sex 5+yrs older (vs ≤4yrs) | 1.74 (0.81-3.77) | 2.09*** (1.22-3.59) | 2.01*** (1.23-3.29) | 1.29 (0.78-2.12) | 1.54* (0.93-2.55) |
| Age | 1.04 (0.86-1.25) | 1.23*** (1.09-1.39) | 1.22*** (1.07-1.39) | 1.13** (1.01-1.26) | 1.20 (0.97-1.24) |
| Non-black African (vs black African) | 0.40* (0.14-1.14) | 0.03*** (0.01-0.08) | 0.11*** (0.05-0.27) | 0.07*** (0.03-0.18) | 0.10*** (0.03-0.27) |
| Married (vs unmarried) | 0.75 (0.21-2.74) | 0.33** (0.14-0.81) | 0.32** (0.12-0.87) | 0.46 (0.14-1.47) |  |
| Marital status (ref = single) |  |  |  |  |  |
| Married |  |  |  |  | 0.71 (0.21-2.44) |
| Cohabitating | - |  |  |  | 2.12** (1.08-4.17) |
| Locality (ref = urban formal) | |  |  |  |  |
| Urban informal | 1.25 (0.47-3.31) | 1.76* (0.96-3.22) | 1.46 (0.78-2.73) | 1.27 (0.68-2.39) | 1.28 (0.64-2.56) |
| Rural informal | 0.41 (0.13-1.23) | 0.86 (0.50-1.49) | 0.97 (0.53-1.77) | 1.27 (0.76-2.11) | 1.54 (0.91-2.60) |
| Rural formal | 1.34 (0.37-4.89) | 1.02 (0.46-2.30) | 1.68 (0.80-3.54) | 1.54 (0.80-3.00) | 1.50 (0.76-2.97) |
| Employment status (ref = unemployed) | |  |  |  |  |
| Student | 0.40 (0.13-1.23) | 1.48 (0.74-2.94) | 0.61 (0.30-1.24) | 0.46** (0.23-0.94) | 0.40*** (0.21-0.78) |
| Employed | 0.93 (0.37-2.35) | 0.97 (0.42-2.24) | 0.90 (0.40-2.04) | 0.79 (0.41-1.51) | 1.17 (0.54-2.53) |
| Condom used at last sex (vs not used) | 1.09 (0.47-2.52) | 0.96 (0.56-1.65) | 1.28 (0.74-2.22) | 2.11*** (1.36-3.27) | 2.70*** (1.72-4.22) |
| Age of first sex ≤15 (vs aged 16+) | 1.05 (0.41-2.71) | 1.14 (0.64-2.04) | 1.45 (0.80-2.62) | 1.48 (0.83-2.66) | 1.55 (0.78-3.05) |
| Multiple sexual partners in past year (vs 1 partner) | 1.78 (0.63-5.04) | 3.33*** (1.60-6.95) | -1.44 (0.57-3.63) | 1.64 (0.83-3.24) | - |
| Number of lifetime partners (ref = 1) | |  |  |  |  |
| 2 lifetime partners | - | - | - | - | 0.87 (0.46-1.63) |
| 3-4 lifetime partners | - | - | - | - | 1.61 (0.85-3.04) |
| 5+ lifetime partners | - | - | - | - | 1.09 (0.41-2.91) |
| Think partner had other partners (vs did not think) | - | - | - | - | 1.95** (1.17-3.27) |
| Income last month >R4000 (vs ≤R4000) | - | - | - | - | 0.06*** (0.01-0.41) |

Note: *=p<0.10; **=p<0.05; and ***=p<0.01

All analyses were adjusted to account for the complex study design (i.e. stratification and clustering) and for non-response (by using weighted data).

**Table S7.** Multiple regression analyses showing associations between years age difference between partners (most recent partner only) and covariates with HIV status for young women aged 15 to 24 years, South Africa 2002, 2005, 2008, and 2012.

|  | **2002 (A)**  **N=446** | **2005 (A)**  **N=994** | **2008 (A)**  **N=854** | **2012 (A)**  **N=1394** | **2012 (B)**  **N=1257** |
| --- | --- | --- | --- | --- | --- |
|  | **aOR (95% CI)** | **aOR (95% CI)** | **aOR (95% CI)** | **aOR (95% CI)** | **aOR (95% CI)** |
| Years age difference (continuous) | 1.09 (0.97-1.22) | 1.10*** (1.03-1.17) | 1.07** (1.00-1.15) | 1.09** (1.02-1.16) | 1.08** (1.01-1.15) |
| Age | 1.04 (0.87-1.25) | 1.22*** (1.08-1.37) | 1.21*** (1.06-1.37) | 1.12** (1.01-1.25) | 1.10 (0.97-1.24) |
| Non-black African (vs black African) | 0.37* (0.12-1.18) | 0.03*** (0.01-0.08) | 0.11*** (0.04-0.27) | 0.07*** (0.03-0.18) | 0.10*** (0.03-0.28) |
| Married (vs unmarried) | 0.71 (0.22-2.30) | 0.33** (0.13-0.80) | 0.32** (0.12-0.86) | 0.41 (0.12-1.34) |  |
| Marital status (ref = single) | |  |  |  |  |
| Married |  |  |  |  | 0.67 (0.20-2.28) |
| Cohabitating | - |  |  |  | 1.87* (0.94-3.69) |
| Locality (ref = urban formal) | |  |  |  |  |
| Urban informal | 1.22 (0.46-3.24) | 1.64 (0.89-3.00) | 1.51 (0.81-2.78) | 1.22 (0.64-2.31) | 1.23 (0.61-2.49) |
| Rural informal | 0.42* (0.16-1.12) | 0.80 (0.46-1.40) | 0.98 (0.53-1.79) | 1.22 (0.74-2.03) | 1.46 (0.86-2.47) |
| Rural formal | 1.36 (0.37-4.90) | 0.94 (0.42-2.11) | 1.65 (0.80-3.41) | 1.51 (0.78-2.93) | 1.48 (0.75-2.93) |
| Employment status (ref = unemployed) | |  |  |  |  |
| Student | 0.40 (0.13-1.23) | 1.46 (0.74-2.91) | 0.59 (0.42-2.08) | 0.48** (0.24-0.99) | 0.41*** (0.21-0.79) |
| Employed | 0.93 (0.36-2.41) | 0.94 (0.40-2.24) | 0.93 (0.42-2.08) | 0.80 (0.41-1.55) | 1.13 (0.52-2.46) |
| Condom used at last sex (vs not used) | 1.11 (0.48-2.53) | 0.98 (0.57-1.68) | 1.23 (0.70-2.16) | 2.19*** (1.43-3.37) | 2.67*** (1.70-4.18) |
| Age of first sex ≤15 (vs 16+) | 1.03 (0.38-2.84) | 1.14 (0.65-1.99) | 1.44 (0.80-2.58) | 1.55 (0.80-3.02) | 1.51 (0.77-2.96) |
| Multiple sexual partners in past year (vs 1 partner) | 1.76 (0.62-4.95) | 3.44*** (1.64-7.23( | 1.48 (0.60-3.65) | 1.44 (0.80-2.60) | - |
| Number of lifetime partners (ref = 1 partner) | |  |  |  |  |
| 2 lifetime partners | - | - | - | - | 0.85 (0.45-1.61) |
| 3-4 lifetime partners | - | - | - | - | 1.55 (0.82-2.92) |
| 5+ lifetime partners | - | - | - | - | 1.09 (0.42-2.81) |
| Think partner had other partners (vs did not think) | - | - | - | - | 1.96** (1.17-3.28) |
| Income last month >R4000 (vs ≤R4000) | - | - | - | - | 0.06*** (0.01-0.43) |

Note: *=p<0.10; **=p<0.05; and ***=p<0.01

All analyses were adjusted to account for the complex study design (i.e. stratification and clustering) and for non-response (by using weighted data).

**Sensitivity analysis considering black African women only**

**Table S8.** Bivariate regression analyses showing associations between age-disparate sex (5+ years older) and years age difference between partners (continuous) and HIV status for young black African women aged 15 to 24 years, South Africa 2002, 2005, 2008, and 2012.

|  | **2002 OR (95% CI)**  **N=350** | **2005 OR (95% CI)**  **N=803** | **2008 OR (95% CI)**  **N=661** | **2012 OR (95% CI)**  **N=1116** |
| --- | --- | --- | --- | --- |
| Age-disparate sex 5+yrs older (vs age similar ≤4yrs) | 2.03* (0.96-4.26) | 2.30*** (1.39-3.80) | 1.88** (1.17-3.03) | 1.22 (0.74-2.01) |
| Years age difference between partners (continuous) | 1.13** (1.02-1.26) | 1.10*** (1.04-1.17) | 1.07** (1.00-1.15) | 1.08** (1.01-1.15) |

Note: *=p<0.10; **=p<0.05; and ***=p<0.01

All analyses were adjusted to account for the complex study design (i.e. stratification and clustering) and for non-response (by using weighted data).

**Table S9.** Multiple regression analyses showing associations between age-disparate sex (5+ years older) and covariates with HIV status for young black African women aged 15 to 24 years, South Africa 2002, 2005, 2008, and 2012.

|  | **2002 (A)**  **N=309** | **2005 (A)**  **N=746** | **2008 (A)**  **N=642** | **2012 (A)**  **N=1039** | **2012 (B)**  **N=939** |
| --- | --- | --- | --- | --- | --- |
|  | **aOR (95% CI)** | **aOR (95% CI)** | **aOR (95% CI)** | **aOR (95% CI)** | **aOR (95% CI)** |
| Years age difference (continuous) | 2.39** (1.01-5.64) | 2.11*** (1.21-3.68) | 1.97*** (1.20-3.24) | 1.24 (0.75-2.06) | 1.48 (0.88-2.46) |
| Age | 1.13 (0.91-1.40) | 1.24*** (1.09-1.40) | 1.22***(1.06-1.39) | 1.12** (1.00-1.25) | 1.09 (0.96-1.23) |
| Married (vs unmarried) | 0.77 (0.19-3.14) | 0.32** (0.13-0.80) | 0.33** (0.12-0.91) | 0.47 (0.14-1.53) |  |
| Marital status (ref = single) | |  |  |  |  |
| Married |  |  |  |  | 0.74 (0.22-2.54) |
| Cohabitating |  |  |  |  | 2.22** (1.11-4.44) |
| Locality (ref = urban formal) | |  |  |  |  |
| Urban informal | 1.06 (0.38-2.89) | 1.79* (0.97-3.28) | 1.42 (0.75-2.69) | 1.28 (0.68-2.41) | 1.31 (0.65-2.66) |
| Rural informal | 0.35** (0.13-0.92) | 0.87 (0.50-1.50) | 0.96 (0.52-1.76) | 1.27 (0.76-2.11) | 1.55 (0.92-2.62) |
| Rural formal | 0.80 (0.21-3.03) | 1.03 (0.46-2.33) | 1.64 (0.77-3.52) | 1.48 (0.73-2.98) | 1.55 (0.76-3.15) |
| Employment status (ref = unemployed) | |  |  |  |  |
| Student | 0.47 (0.15-1.46) | 1.47 (0.74-2.90) | 0.60 (0.29-1.25) | 0.46** (0.22-0.94) | 0.40*** (0.20-0.78) |
| Employed | 0.43 (0.15-1.28) | 0.96 (0.40-2.29) | 0.91 (0.38-2.16) | 0.82 (0.42-1.57) | 1.22 (0.56-2.68) |
| Condom used at last sex (vs not used) | 1.03* (0.41-2.58) | 0.97 (0.56-1.68) | 1.24 (0.71-2.18) | 2.06*** (1.32-3.21) | 2.62*** (1.66-4.12) |
| Age of first sex ≤15 (vs 16+) | 0.83 (0.31-2.21) | 1.18 (0.66-2.11) | 1.45 (0.79-2.66) | 1.46 (0.81-2.66) | 1.52 (0.75-3.05) |
| Multiple sexual partners in past year (vs 1 partner) | 2.64* (0.84-8.26) | 2.95*** (1.36-6.40) | 1.49 (0.58-3.82) | 1.66 (0.84-3.30) |  |
| Number of lifetime partners (ref = 1 partner) | |  |  |  |  |
| 2 lifetime partners | - | - | - | - | 0.82 (0.43-1.57) |
| 3-4 lifetime partners | - | - | - | - | 1.61 (0.85-3.07) |
| 5+ lifetime partners | - | - | - | - | 1.10 (0.41-3.00) |
| Think partner had MSPs (vs did not think) | - | - | - | - | 1.92** (1.14-3.23) |
| Income last month >R4000 (vs ≤R4000) | - | - | - | - | 0.06*** (0.01-0.45) |

Note: *=p<0.10; **=p<0.05; and ***=p<0.01

All analyses were adjusted to account for the complex study design (i.e. stratification and clustering) and for non-response (by using weighted data).

**Table S10.** Multiple regression analyses showing associations between years age difference between partners (continuous) and covariates with HIV status for young black women aged 15 to 24 years, South Africa 2002, 2005, 2008, and 2012.

|  | **2002 (A)**  **N=309** | **2005 (A)**  **N=994** | **2008 (A)**  **N=642** | **2012 (A)**  **N=1039** | **2012 (B)**  **N=939** |
| --- | --- | --- | --- | --- | --- |
|  | **aOR (95% CI)** | **aOR (95% CI)** | **aOR (95% CI)** | **aOR (95% CI)** | **aOR (95% CI)** |
| Years age difference (continuous) | 1.21*** (1.07-1.37) | 1.09*** (1.03-1.16) | 1.08** (1.01-1.16) | 1.08** (1.01-1.16) | 1.08** (1.01-1.15) |
| Age | 1.14 (0.92-1.40) | 1.22*** (1.08-1.38) | 1.21*** (1.05-1.38) | 1.12** (1.00-1.25) | 1.09 (0.96-1.23) |
| Married (vs unmarried) | 0.55 (0.14-2.13) | 0.33** (0.13-0.81) | 0.32** (0.12-0.89) | 0.42 (0.13-1.39) | 0.70 (0.20-2.37) |
| Marital status (ref = single) | |  |  |  |  |
| Married |  |  |  |  |  |
| Cohabitating | - |  |  |  |  |
| Locality (ref = urban formal) | |  |  |  |  |
| Urban informal | 0.94 (0.33-2.73) | 1.65 (0.90-3.03) | 1.45 (0.77-2.73) | 1.23 (0.64-2.34) | 1.26 (0.62-2.57) |
| Rural informal | 0.35** (0.13-0.95) | 0.79 (0.45-1.40) | 0.97 (0.53-1.78) | 1.23 (0.74-2.04) | 1.48 (0.88-2.50) |
| Rural formal | 0.70 (0.20-2.52) | 0.95 (0.42-2.14) | 1.60 (0.76-3.38) | 1.46 (0.72-2.95) | 1.55 (0.76-3.16) |
| Employment status (ref = unemployed) | |  |  |  |  |
| Student | 0.45 (0.14-1.42) | 1.46 (0.73-2.89) | 0.58 (0.28-1.21) | 0.48** (0.23-0.99) | 0.40*** (0.21-0.78) |
| Employed | 0.38* (0.13-1.10) | 0.92 (0.37-2.26) | 0.93 (0.40-2.18) | 0.82 (0.42-1.61) | 1.18 (0.53-2.60) |
| Condom used at last sex (vs not used) | 1.01** (0.41-2.48) | 0.98 (0.57-1.69) | 1.20 (0.67-2.14) | 2.13*** (1.38-3.29) | 2.58*** (1.63-4.07) |
| Age of first sex ≤15 (vs 16+) | 0.65 (0.21-2.02) | 1.17 (0.67-2.06) | 1.45 (0.79-2.64) | 1.43 (0.78-2.60) | 1.49 (0.75-2.98) |
| Multiple sexual partners in past year (vs 1 partner) | 2.71* (0.90-8.17) | 2.91*** (1.33-6.37) | 1.50 (0.60-3.73) | 1.52 (0.77-3.00) |  |
| Number of lifetime partners (ref = 1) | |  |  |  |  |
| 2 lifetime partners | - | - | - | - | 0.80 (0.42-1.54) |
| 3-4 lifetime partners | - | - | - | - | 1.55 (0.82-2.96) |
| 5+ lifetime partners | - | - | - | - | 1.08 (0.41-2.86) |
| Think partner had MSPs (vs did not think) | - | - | - | - | 1.93** (1.15-3.24) |
| Income last month >R4000 (vs ≤R4000) | - | - | - | - | 0.07*** (0.01-0.48) |

Note: *=p<0.10; **=p<0.05; and ***=p<0.01

All analyses were adjusted to account for the complex study design (i.e. stratification and clustering) and for non-response (by using weighted data).
